# Psychosocial factors are associated with community mobility and participation in persons with dizziness

**DOI:** 10.3389/fneur.2025.1531204

**Published:** 2025-01-29

**Authors:** Pamela M. Dunlap, Jeffrey P. Staab, Patrick J. Sparto, Joseph M. Furman, Susan L. Whitney

**Affiliations:** ^1^Department of Physical Therapy, University of Pittsburgh, Pittsburgh, PA, United States; ^2^Department of Psychiatry and Psychology, Otolaryngology Head and Neck Surgery Mayo Clinic, Rochester, MN, United States; ^3^Department of Otolaryngology, University of Pittsburgh, Pittsburgh, PA, United States

**Keywords:** life space, activity limitations, participation restrictions, anxiety, depression, fear avoidance, catastrophizing

## Abstract

**Introduction:**

Among individuals with dizziness, there is an increased prevalence of psychosocial comorbidity compared to the general population. Increased psychosocial comorbidity among people with dizziness is associated with disability and poorer outcomes in vestibular rehabilitation. However, there is less knowledge regarding the association between psychosocial factors and mobility outcomes in people with dizziness. Therefore, the purpose of this study was to assess the association between psychosocial factors and future activity, participation, and community mobility among people with dizziness. The secondary aim of this study was to explore the constructs measured using patient-reported outcomes associated with psychosocial factors in this population.

**Materials and methods:**

We conducted a prospective cohort study with an in-person baseline assessment and a follow-up assessment completed at 3-months via computerized questionnaires. We measured psychosocial factors at baseline using the Hospital Anxiety and Depression Scale (HADS), the Patient Health Questionnaire 4-item (PHQ-4), the Vestibular Activities Avoidance Instrument (VAAI), and the Dizziness Catastrophizing Scale (DCS). We measured community mobility and participation at baseline and 3-month follow-up using the Life Space Assessment (LSA) and Vestibular Activities and Participation Measure (VAP). To determine the associations between baseline anxiety and depression symptoms, fear avoidance, catastrophizing beliefs and mobility and disability measures, we used simple linear regression and repeated measures ANOVA. We used exploratory factor analysis to identify constructs measured using patient-reported outcomes of psychosocial factors.

**Results:**

There were 100 participants who completed the baseline assessment [mean age (SD) = 49.2 (15.7) years; 73% female] and 68 participants completed the questionnaire at the 3-month follow-up. In bivariate analyses, baseline VAAI, HADS-A, HADS-D, PHQ-4 depression, and VAP were associated with LSA, and baseline VAAI, HADS-D, PHQ-4 depression, and LSA were associated with VAP at 3-month follow-up (all *p* < 0.05). In adjusted multivariate analyses, baseline VAP had a large effect (*F* = 11.65, *p* = 0.001, *η*^2^ = 0.18) and HADS-D had a moderately large effect (*F* = 4.09, *p* = 0.048, *η*^2^ = 0.07) on LSA score at 3-month follow-up. Baseline VAAI had a large effect (*F* = 23.35, *p* < 0.001, *η*^2^ = 0.3) on VAP at 3-month follow-up. The exploratory factor analysis of the VAAI, HADS, PHQ-4, and DCS resulted in 4 factors measuring constructs of fear avoidance, anxiety, depression, and catastrophization.

**Discussion:**

We found that baseline psychosocial factors were related to future measures of activity and participation as well as community mobility in people with dizziness. Specifically, baseline activity and participation levels and depressive symptoms were significantly associated with future community mobility and baseline fear avoidance beliefs were significantly associated with future activity and participation. Patient-reported outcome measures of psychosocial factors appear to measure unique constructs, which may indicate that a small number of different outcome measures may be needed to gather important prognostic information to manage individuals with dizziness well.

## Introduction

There is an increased prevalence of psychosocial comorbidity among individuals with dizziness compared to the general population (1, 2). Staab and Ruckenstein (3) described three relationships between dizziness and psychiatric disorders over the course of illness: a primary psychiatric condition causing dizziness, a primary neurotologic condition that triggers a secondary psychiatric disorder, or a primary neurotologic condition that exacerbates a pre-existing psychiatric disorder. In cross-sectional studies, researchers have found that psychosocial factors in persons with dizziness are associated with poorer quality of life, greater dizziness severity, and dizziness-related disability, as well as greater healthcare utilization (2, 4). These findings have been confirmed in prospective analyses where factors such as anxiety, depression, fear avoidance, and illness perceptions were associated with dizziness-related disability and longer recovery times in persons with dizziness (5–8).

Given the impact of psychosocial factors on measures of disability and prognosis in people experiencing dizziness, it is important to incorporate these factors into treatment plans for vestibular rehabilitation. A number of patient-reported outcomes have been used in this population to screen for anxiety and depressive symptoms such as the Hospital Anxiety and Depression Scale (HADS), Patient Health Questionnaire-9, and Generalized Anxiety Disorder Scale-7, among others (9–13). Scales have also been developed to measure additional psychosocial constructs that impact disability in people with dizziness such as fear avoidance beliefs, catastrophization, and illness beliefs (14–16). Although each of these psychosocial factors have been studied and found to be associated with dizziness-related disability in individuals with dizziness, it remains unclear if certain factors play a more critical role in recovery from a vestibular disorder than others and how to best address them in vestibular rehabilitation clinics. Therefore, there is a need to identify which measures may be most beneficial for clinicians to use to assist with treatment plans and determination of prognosis.

While a number of studies have found associations between psychosocial factors and dizziness-related handicap (4, 17, 18), there is a gap in knowledge regarding the association between psychosocial factors and community mobility in persons with dizziness. Therefore, the purpose of this study was to determine the association between psychosocial factors with future community mobility, activity, and participation among persons with dizziness. A secondary aim was to assess the dimensionality of various measures of psychosocial factors that are used in vestibular rehabilitation.

## Materials and methods

### Participants

We recruited a convenience sample of individuals seeking care for dizziness at a tertiary care balance disorders clinic. The inclusion criteria were that individuals were currently experiencing dizziness, 18 years of age or older, English-speaking, and cognitively able to provide written and verbal informed consent. The study was approved by the University of Pittsburgh Institutional Review Board (STUDY20040188). Diagnoses were made by a oto-neurologist and were categorized as follows: peripheral vestibular hypofunction, benign paroxysmal positional vertigo (BPPV), mixed peripheral vestibular hypofunction and BPPV, Meniere’s Disease, vestibular migraine, mixed vestibular migraine and peripheral vestibular hypofunction, persistent postural perceptual dizziness (PPPD), other central vestibular disorders, imbalance or gait disorders, and unspecified dizziness.

### Baseline and three-month follow-up assessments

Participants completed an in-person baseline assessment at the tertiary care balance disorders clinic, which included a computerized questionnaire of demographic questions, symptom self-reports, and patient-reported outcome measures. Participants were asked if they would like to be contacted via phone or email in 3 months. Based on their selection, they were either contacted via phone to verbally complete the questionnaires or by email to complete the questionnaires using a survey link. A follow-up time of 3 months was selected because the purpose of our study was to assess the effects of baseline psychosocial factors on future community mobility and participation, and 3 months after the onset of dizziness symptoms are generally considered to be in the chronic phase where symptoms from an acute vestibulopathy would either have recovered or persisted to the chronic phase (19).

### Outcome measures

#### Community mobility and participation

Community mobility was assessed using the Life Space Assessment (LSA), which is a measure of how much and how often an individual moves within their environment and also takes into account assistance needed for mobility (20). The LSA is a self-report measure of mobility over the past month and ranges from a total score of 0 indicating that the person did not move outside of their bedroom to 120 indicating that the person frequently moves outside of their town without assistance. The LSA has excellent reliability and is correlated with dizziness-related disability and quality of life in persons with dizziness (21). In this study, the LSA was used as a dependent variable for our multivariate analysis of the association between psychosocial factors and community mobility.

Activity and participation was measured using the Vestibular Activities and Participation Measure (VAP) (22). The VAP was developed based on the International Classification of Functioning, Disability and Health and can identify activity limitations and participation restrictions among persons with dizziness (22, 23). The VAP total score ranges from 0, which indicates no activity limitations or participation restrictions to 4, which indicates complete limitations in activity and participation. The VAP has excellent reliability and is correlated with quality of life and dizziness-related disability among individuals with dizziness (22). In this study, the VAP was used as a dependent variable for our multivariate analysis of the association between psychosocial factors and activity and participation.

#### Psychosocial factors

We used several patient-reported outcome measures to measure various psychosocial constructs (i.e., anxiety and depression, fear avoidance, and catastrophization). The HADS was used to measure anxiety and depressive symptoms. The HADS was developed as a screening tool for clinically significant anxiety and depression in primary care settings (9). The HADS consists of an anxiety subscale (HADS-A) and depression subscale (HADS-D), which both range from 0 to 21. A score of 8–10 on either subscale is associated with possible clinically significant anxiety or depression and a score of 11 or greater is associated with probable clinically significant anxiety or depression (9). The HADS has been used in samples of individuals with dizziness and has moderate to strong correlations with dizziness-related disability (12, 18).

We also used the Patient Health Questionnaire for Anxiety and Depression (PHQ-4) as a second measure of anxiety and depressive symptoms. The PHQ-4 was developed as an ultra-brief screening tool for anxiety and depressive symptoms and includes two subscales that each range from 0 to 6 with a score of 3 or greater indicating a possible underlying anxiety and depressive disorder (24).

The Vestibular Activities Avoidance Instrument (VAAI) was developed to identify fear avoidance beliefs and avoidance of activity among persons with dizziness (14). The VAAI consists of 9 items and the total score ranges from 0 to 54 with higher scores indicating greater fear avoidance beliefs and behaviors. The VAAI has excellent reliability and moderate to strong associations with measures of disability among individuals with dizziness (7, 25).

The Dizziness Catastrophizing Scale (DCS) was adapted for individuals experiencing dizziness from the Pain Catastrophizing Scale to measure the construct of catastrophization, which is a maladaptive thought process that amplifies negative assessments of symptoms and anticipates worst case scenario outcomes (15). The DCS has excellent reliability and there is evidence for validity in persons with dizziness (15).

#### Covariates

We included age, gender, number of medications, and dizziness severity measures using a visual analogue scale (VAS) as covariates. Age was determined by the difference between the participant’s date of birth and baseline visit date. Gender was self-reported (female, male, other). Number of medications was determined upon review of the electronic health record for current medications. Participants rated their dizziness over the last 24 h using a visual analogue scale from 0 to 10 with 10 being the most severe dizziness (26).

### Statistical analyses

We used descriptive statistics (proportion, mean, standard deviation) to assess demographic characteristics and baseline and 3-month outcome measure scores. We used simple linear regression to identify the bivariate associations between baseline psychosocial factors and 3-month community mobility and participation measures. We reported R squared values, unstandardized beta coefficients, standard error, and *p*-values from the simple linear regression models. Variables that were significant (*p* < 0.05) were entered into two repeated measures ANOVA models to assess their multivariate relationships to changes in LSA and VAP scores from baseline to 3 months. We included the following covariates based on our previous work: age, gender, number of medications, and dizziness severity (7). We assessed for multicollinearity between predictors using Spearman’s correlation coefficient and identified 0.80 as evidence for multicollinearity (27). We reported the F statistic, *p* value, and partial eta squared effect sizes to determine the strength of associations using the following thresholds suggested by Cohen (28) small effect = 0.01, medium effect = 0.06, large effect = 0.14.

To identify key dimensions of behavioral morbidity contained within psychosocial measures (HADS, PHQ-4, VAAI, and DCS) that may be relevant to patients with dizziness, we performed principal component exploratory factor analysis. First, we evaluated Bartlett’s test of sphericity for significance and the Kaiser-Meyer-Olkin measure of sampling adequacy (>0.6) to determine if the items were factorable (29, 30). To determine the number of factors to retain, we evaluated the scree plot for a point of inflection. We then used direct oblimin rotation and reported the factor loadings from the factor structure matrix, which represent simple zero-order correlations of the items, where the size of the loading is not influenced by the correlation between factors (30). We suppressed factor loadings <0.4 to interpret the factors and identify patterns of the item to factor correlations.

## Results

### Sample description

At baseline, the average age of the sample of 100 participants experiencing dizziness was 49.2 (15.7) years and 73% were female (Table 1). Approximately one-third of the sample were diagnosed with unspecified dizziness (31%), followed by peripheral vestibular hypofunction (27%), mixed vestibular migraine and peripheral vestibular hypofunction (10%), BPPV (8%), and vestibular migraine. Most participants were experiencing chronic symptoms (3 months or greater since symptom onset) with 31% experiencing acute symptoms (*n* = 6 were missing data for this variable). The average HADS-A was 8.0 (4.4) and the PHQ-4 anxiety subscale was 4.1 (1.9) at baseline, indicating that the study cohort as a whole had possible clinically significant anxiety, but minimal depressive symptoms. The average baseline VAP score was 1.0 (0.7), indicating mild activity limitations and participation restrictions overall, which significantly improved at the 3-month follow-up visit to 0.8 (0.7) (*p* < 0.001). The average baseline LSA score was 74.3 (25.8), indicating restricted community mobility, which did not improve at the 3-month follow-up visit [76.1 (26.1)].

**Table 1:** Characteristics and patient-reported outcome measure scores for 100 participants with dizziness.

	Baseline (*n* = 100) Mean (SD) or *n* (%)	3-Month Follow-up (*n* = 68) Mean (SD)
Age	49.2 (15.7)	
Female	73 (73)	
Acute condition (*n* = 94)	29 (30.9)	
Diagnosis		
Peripheral vestibular hypofunction	27 (27)	
Benign paroxysmal positional vertigo (BPPV)	8 (8)	
Mixed BPPV and Peripheral vestibular hypofunction	6 (6)	
Meniere’s Disease	3 (3)	
Vestibular migraine	7 (7)	
Mixed vestibular migraine and peripheral vestibular hypofunction	10 (10)	
Persistent postural perceptual dizziness	4 (4)	
Other central vestibular disorders	2 (2)	
Imbalance or gait disturbance	2 (2)	
Unspecified dizziness	31 (31)	
Dizziness visual analogue scale (0–10)	4.0 (2.5)	
Number of medications	7.8 (5.3)	
Vestibular activities avoidance instrument (0–54)	25.4 (13.3)	
Dizziness catastrophizing scale (0–52)	16.0 (12.2)	
Hospital anxiety and depression scale—anxiety (0–21)	8.0 (4.4)	
Hospital anxiety and depression scale—depression (0–21)	4.5 (2.8)	
Patient health questionnaire—anxiety (0–6)	4.1 (1.9)	
Patient health questionnaire—depression (0–6)	3.3 (1.5)	
Vestibular activities and participation measure (0–4)*	1.0 (0.7)	0.8 (0.07)
Life space assessment (0–120)	74.3 (25.8)	76.1 (26.1)

^*^Significant difference between baseline and 3-month outcome measure scores (*p* < 0.001).

### Association between psychosocial factors and community mobility

We found significant bivariate associations in simple linear regression models between baseline VAAI, HADS-A, HADS-D, PHQ-4 depression subscale, and LSA score at the 3-month follow-up (Table 2). There was also a significant relationship between baseline VAP and LSA score at 3 months. Therefore, we included these measures in the repeated measures ANOVA model for LSA. Upon assessing the association between the independent variables and covariates, we found no correlation coefficients >0.8, providing evidence for lower risk of multicollinearity (27). After controlling for age, gender, dizziness severity, number of medications as well as baseline VAAI, HADS-A, PHQ-4 depression subscale and LSA, we found that the baseline HADS-D (*F* = 4.09, *p* = 0.048) and VAP (*F* = 11.65, *p* = 0.001) were significantly associated with the total LSA score at the 3-month follow-up visit (Table 3). This indicates that baseline depressive symptoms and activity limitations and participation restrictions were significant predictors of community mobility at 3 months. According to the partial eta squared values, baseline VAP had a large effect (*η*^2^ = 0.18), and HADS-D had a moderate to large effect (*η*^2^ = 0.07) on community mobility at 3 months.

**Table 2:** Association between baseline psychosocial factors and community mobility and participation at follow-up.

Dependent variable: follow-up life space assessment				
Independent variable	R square	Unstandardized β	Standard error	*p*-value
Dizziness catastrophizing scale	0.0	−0.03	0.27	0.926
Hospital anxiety and depression scale—anxiety	0.10	−2.80	1.05	0.010*
Hospital anxiety and depression scale—depression	0.11	−5.24	1.83	0.006*
Patient health questionnaire—anxiety	0.04	−2.68	1.67	0.113
Patient health questionnaire—epression	0.11	−5.34	1.83	0.006*
Vestibular activities avoidance instrument	0.06	−0.47	0.23	0.041*
Vestibular activities and participation measure	0.24	−19.4	4.23	<0.001*
Dependent variable: follow-up vestibular activities and participation measure				
Dizziness catastrophizing scale	0.05	0.01	0.01	0.063
Hospital anxiety and depression scale—anxiety	0.04	0.03	0.02	0.093
Hospital anxiety and depression scale—depression	0.11	0.08	0.03	0.006*
Patient health questionnaire—anxiety	0.02	0.05	0.05	0.250
Patient health questionnaire—depression	0.12	0.15	0.05	0.003*
Life space assessment	0.13	−0.01	0.003	0.002*
Vestibular activities avoidance instrument	0.32	0.03	0.01	<0.001*

*p < 0.05.

**Table 3:** Predictors of community mobility at follow-up among individuals with dizziness.

Variable	F	*p*-value	Partial eta squared
Hospital anxiety and depression scale–anxiety	3.55	0.065	0.063
Hospital anxiety and depression scale–depression	4.09	0.048*	0.072
Life space assessment	0.27	0.608	0.005
Patient health questionnaire–depression	3.59	0.064	0.063
Vestibular activities avoidance instrument	3.92	0.053	0.069
Vestibular activities and participation measure	11.65	0.001*	0.180

^*^p < 0.05. Dependent variable was follow-up life space assessment score; Model was adjusted for age, gender, number of medications, and dizziness severity.

### Association between psychosocial factors and activity and participation

There were significant bivariate associations between baseline VAAI, HADS-D, PHQ-4 depression subscale, and the total VAP score at the 3-month follow-up visit (Table 2). We also found a significant association between baseline LSA score and VAP score at 3 months. We included each of these baseline measures as well as baseline VAP into the repeated measures ANOVA model of VAP. Upon assessing the association between the independent variables and covariates, we found no correlation coefficients >0.8, providing evidence for lower risk of multicollinearity (27). We found that baseline VAAI was the only significant predictor of total VAP score at 3-month follow-up after controlling for covariates and other psychosocial factors (*F* = 23.35, *p* < 0.001) (Table 4). This indicates that baseline fear avoidance beliefs were a significant predictor of future activity limitations and participation restrictions. According to the partial eta squared value, baseline VAAI had a large effect (η_2_ = 0.3) on VAP at the 3-month follow-up visit.

**Table 4:** Predictors of activity and participation at follow-up among individuals with dizziness.

Variable	F	*p*-value	Partial eta squared
Hospital anxiety and depression scale—depression	0.40	0.529	0.007
Life space assessment	1.39	0.243	0.025
Patient health questionnaire—depression	1.89	0.175	0.034
Vestibular activities avoidance instrument	23.35	<0.001*	0.302
Vestibular activities and participation measure	0.90	0.346	0.016

^*^p < 0.05. Dependent variable was follow-up Vestibular Activities and Participation Measure score; Model was adjusted for age, gender, number of medications, and dizziness severity.

### Exploratory factor analysis of psychosocial outcome measures

According to Bartlett’s test of sphericity (Χ^2^ = 2935, *p* < 0.001) and the KMO measure of sampling adequacy (KMO = 0.852), there was a sufficient number of correlated items to conduct the factor analysis. Upon evaluation of the scree plot (Supplementary Figure 1), we found that four factors should be retained. After the direct oblimin factor rotation, most items strongly loaded onto at least one factor (Table 5). Based on the factor loadings, we named the factors as follows: Catastrophization (factor 1), Anxiety (factor 2), Fear Avoidance (factor 3), and Depression (factor 4). One item from the HADS depression subscale did not load strongly on any factor. This item was related to losing interest in appearance. HADS item 8, or feeling slowed down, loaded on the fear avoidance factor. Item 6 from the HADS depression subscale, relating to being cheerful, and Item 4 from the PHQ-4 relating to feeling down, both loaded more strongly on the anxiety factor than the depression factor.

**Table 5:** Factor loadings for psychosocial measure items among individuals with dizziness.

Measure and item	Factor 1 catastrophization	Factor 2 anxiety	Factor 3 fear avoidance	Factor 4 depression
VAAI 1: strenuous housework difficult			0.796	
VAAI 2: cannot do physical activities			0.771	
VAAI 3: dizziness interferes with responsibilities			0.839	
VAAI 4: participation is restricted			0.758	
VAAI 5: afraid of dizziness with exercise			0.713	
VAAI 6: should not do work			0.732	
VAAI 7: cannot do things normal people do			0.880	
VAAI 8: afraid to leave home			0.667	
VAAI 9: work makes dizziness worse			0.615	
HADS 1: feeling tension		0.825		
HADS 2: enjoyment*				0.512
HADS 3: frightened feeling	0.537	0.719		
HADS 4: laughter				0.656
HADS 5: worrying thoughts	0.406	0.852		
HADS 6: cheerful feeling		0.726		0.435
HADS 7: relaxed feeling		0.684		0.505
HADS 8: feeling slowed down			0.616	
HADS 9: frightened feeling		0.630		
HADS 10: lost interest in appearance*				
HADS 11: restless feeling		0.534		
HADS 12: look forward to things				0.709
HADS 13: feeling of panic		0.831		
HADS 14: enjoyment of book/TV				0.514
PHQ 1: feeling nervous		0.840		0.414
PHQ 2: uncontrolled worrying		0.859		
PHQ 3: loss of interest		0.520		0.627
PHQ 4: feeling down		0.680		0.485
DCS 1: worry if dizziness will end	0.775		0.470	
DCS 2: cannot go on	0.428			
DCS 3: never going to get better	0.810			
DCS 4: overwhelming	0.802	0.402	0.546	
DCS 5: cannot stand it anymore	0.748	0.429		
DCS 6: afraid dizziness will worsen	0.787			
DCS 7: thinking of other dizziness events	0.751	0.458		
DCS 8: anxiously want it to go away	0.794			
DCS 9: cannot keep it out of my mind	0.881			
DCS 10: troublesome	0.868	0.470	0.444	
DCS 11: want it to stop badly	0.886			
DCS 12: cannot reduce intensity	0.630			
DCS 13: something serious may happen	0.651			

^*^Item did not load strongly on any factor. VAAI, Vestibular Activities Avoidance Instrument; HADS, Hospital Anxiety and Depression Scale; PHQ, Patient Health Questionnaire-4; DCS, Dizziness Catastrophizing Scale.

## Discussion

Our findings indicate that various psychosocial factors were associated with future community mobility, activity, and participation. In multivariate models, current depression and activity/participation were significant predictors of future community mobility while fear avoidance was a significant predictor of future activity and participation. Additionally, our results further validate the use of well-established patient-reported outcome measures of psychosocial factors in vestibular rehabilitation in their original formats as they assess several distinct underlying constructs, highlighting the value in employing multiple measures to comprehensively capture psychosocial domain in clinical practice.

### Psychosocial factors, community mobility, activity and participation

In this sample of individuals experiencing dizziness we found limited community mobility evidenced by the total LSA score, which is consistent with what we have found previously among persons with dizziness (21). Our findings suggest that greater depressive symptoms and more activity limitations and participation restrictions were significantly associated with limited future community mobility. Previous studies have found associations between psychosocial factors, including depression, and community mobility among older adults (31, 32). Webber et al. (31) included measures of anxiety, depression, loneliness, social support and frequency of community-based activity and participation in the psychosocial component of their model, which was directly associated with community mobility in older adults. In a study of individuals post-vestibular schwannoma resection, researchers found that individuals with chronic dizziness had lower physical activity levels compared to individuals without chronic dizziness and physical activity levels were significantly associated with disability (33). In our previous work we found that reduced community mobility was associated with greater dizziness-related disability and poorer physical and mental quality of life (21). Together, these findings suggest that psychosocial factors such as depressive symptoms and behavioral variables such as lower physical activity levels may adversely affect community mobility and overall quality of life.

Overall, individuals in our sample reported mild activity limitations and participation restrictions at baseline, which improved over 3 months. Fear avoidance beliefs were significantly associated with future activity limitations and participation restrictions after controlling for other factors. In a prospective study including individuals with acute unilateral vestibulopathy, investigators found that fear avoidance and depressive symptoms at baseline were significantly associated with dizziness-related disability at 6 months post-onset (6). Others found cross-sectional associations between psychosocial factors such as distress, symptom focusing, embarrassment avoidance, resting behaviors, fear avoidance beliefs/behaviors and dizziness-related disability (4, 17, 34). In a separate sample of individuals with dizziness, we previously found significant associations between fear avoidance and activity and participation after controlling for other factors, confirming our findings in the present study.

The goals of vestibular rehabilitation are to minimize symptoms and maximize functioning in activities of daily living, normalize balance and gait, and meet the specific needs and goals of the patient (19). The results of this study suggest that identification of psychological factors such as fear avoidance, catastrophic thinking, and clinically significant depression may allow clinicians to incorporate psychologically informed techniques into rehabilitation plans and collaborate with psychologists and psychiatrists when needed to improve patient outcomes. Self-report measures such as those employed in this investigation are simple, validated aids for detecting these factors. Evidence is beginning to accumulate about the benefits of this approach (35). Psychologically informed techniques in vestibular rehabilitation can include graded exposure to reduce fear of movement, goal-setting, promoting self-efficacy, positive reinforcement, enhancing patient perceptions and expectations, distraction and/or relaxation techniques (36–38). To effectively incorporate these techniques into rehabilitation sessions early on in care, it is essential to identify and measure psychosocial factors in this population. Use of psychologically informed care may address some of the psychosocial factors that are associated with reduced activity, participation, and community mobility among individuals with dizziness.

### Psychosocial constructs measured using patient-reported outcomes

Clinicians have a variety of patient-reported outcome measures available to assess and characterize psychosocial factors among individuals experiencing dizziness. The goal of our factor analysis was to identify distinct constructs relevant to patients with dizziness that may be embedded within these tools. The analysis revealed four factors, which we named catastrophization, anxiety, fear avoidance, and depression. Most items strongly loaded onto one factor in keeping with the construction of the original instruments, further validating the use of the psychosocial measures in this population. However, it should be noted that there was one item that did not load strongly onto any factor and two items from the HADS and PHQ depression subscales that loaded more strongly on the anxiety factor.

Previous studies have examined the factor structure of the HADS, DCS, and VAAI but this is the first study that considers these items together in a factor analysis (12, 14, 15). Piker et al. (12) found a 3-factor structure for the HADS in a sample of individuals with dizziness. Pothier et al. (15) found that the items included in the DCS loaded on a single factor, which was consistent with our findings. In our previous work using a separate sample of individuals with dizziness, we found that the VAAI loaded onto a single factor, confirmed by the results of the present study (14). Taken together, the results of the factor analysis indicate that the DCS, the VAAI, the HADS and PHQ anxiety and depression subscales are all applicable to patients with dizziness without modification, but they also suggest that each captures a different dimension of behavioral morbidity. Thus, there may be benefits in selecting multiple measures to fully capture psychosocial constructs such as catastrophizing, anxiety, fear avoidance, and depression. Given our findings of associations between fear avoidance and depression with activity, participation, and community mobility, these constructs may be particularly important to assess in clinical settings.

### Strengths and limitations

This study included 100 individuals experiencing dizziness recruited from one tertiary care balance disorders clinic. While this sample size was sufficient to include patients with a variety of illnesses, it may not provide an accurate representation of all individuals seeking care for dizziness. We included several patient-reported outcome measures to characterize psychosocial factors, but we did not presume to capture all aspects of psychosocial functioning and there may be other constructs that contribute to activity, participation, and community mobility but were not measured in this study. We were not able to determine reasons that patients were lost to follow-up at 3-months (*n* = 32) as we were not able to contact these individuals, so we cannot determine the extent to which that may have introduced non-random selection bias. Despite these limitations, the results of our multivariate analyses fills a gap in knowledge about the effects of psychosocial factors on activity, participation, and community mobility in this population.

### Conclusion

In this investigation of psychosocial factors in patients experiencing dizziness, we found that depressive symptoms and activity/participation restrictions emerged as predictors of future community mobility, while fear avoidance beliefs strongly predicted future activity limitations and participation restrictions. A possible implication of these results, to be tested in future investigations, is that timely identification of these factors followed by interventions to address them in patients with dizziness may improve outcomes in physical therapy to include greater engagement in activities and increased community mobility.

## Data Availability

The raw data supporting the conclusions of this article will be made available by the authors, without undue reservation.

## Supplementary material

Supplemental Figure 1 Scree plot for exploratory factor analysis of psychosocial outcome measures in people with dizziness.
