# Supplementary figures and images for: Psychosocial factors are associated with community mobility and participation in persons with dizziness
Source: Front Neurol. 2025 Jan 29;16:1531204. doi: 10.3389/fneur.2025.1531204 (PMC11813757; doi:10.3389/fneur.2025.1531204)

**Supplemental Figure 1**


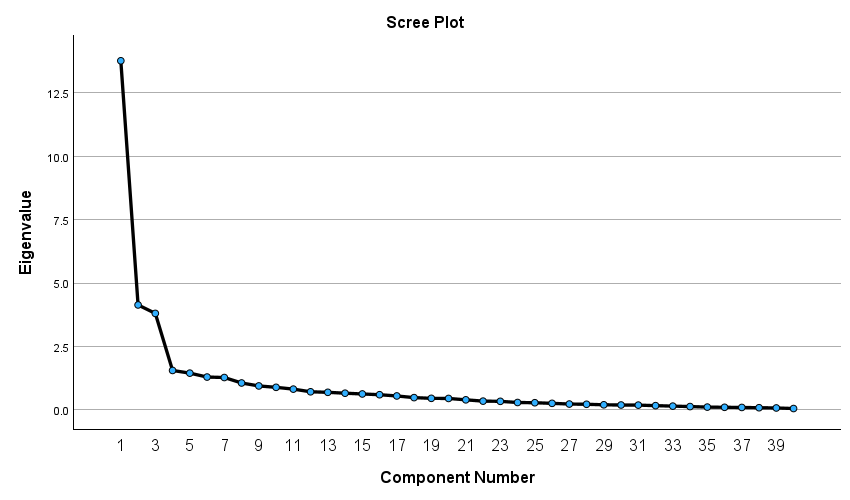

Supplement: Supplemental Figure 1 — Scree plot for exploratory factor analysis of psychosocial outcome measures in people with dizziness. [file Table_1.docx]
